# Circulating extracellular vesicles containing S100A9 reflect histopathology, immunophenotype and therapeutic responses of liver metastasis in colorectal cancer patients

**DOI:** 10.1038/s44276-023-00007-9

**Published:** 2023-08-02

**Authors:** Migmar Tsamchoe, Anthoula Lazaris, Diane Kim, Lucyna Krzywon, Jessica Bloom, Thomas Mayer, Stephanie K Petrillo, Kurt Dejgaard, Zu-Hua Gao, Janusz Rak, Peter Metrakos

**Affiliations:** 1 Department of Anatomy and Cell Biology, McGill University, Montreal, QC Canada; 2 Research Institute of McGill University Health Center, Cancer Research Program, Montreal, QC Canada; 3 Department of Biochemistry, McGill University, Montreal, QC Canada; 4 Department of Pathology and Laboratory Medicine, University of British Columbia, Vancouver, BC Canada; 5 McGill University, Montreal Children’s Hospital, RI MUHC, Montreal, QC Canada; 6 Department of Surgery, McGill University Health Center, Montreal, QC Canada

## Abstract

**Background:**

Metastasis is the principal cause of cancer treatment failure and an area of dire diagnostic needs. Colorectal cancer metastases to the liver (CRCLMs) are predominantly classified into desmoplastic and replacement based on their histological growth patterns (HGPs). Desmoplastic responds well to current treatments, while replacement HGP has a poor prognosis with low overall survival rates.

**Methods:**

We hypothesised that complex cellular response underlying HGPs may be reflected in the proteome of circulating extracellular vesicles (EVs). EV proteomics data was generated through LC-MS/MS and analysed with Maxquant and Perseus. To validate the S100A9 signature, ELISA was performed, and IHC and IF were conducted on tissue for marker detection and colocalization study.

**Results:**

Plasma EV proteome signature distinguished desmoplastic from the replacement in patients with 22 differentially expressed proteins, including immune related markers. Unsupervised PCA analysis revealed clear separation of the two lesions. The marker with the highest confidence level to stratify the two HGPs was S100A9, which was traced in CRCLM lesions and found to colocalize with macrophages and neutrophils. EV-associated S100A9 in plasma may reflect the innate immunity status of metastatic lesions and their differential therapeutic responses.

**Conclusion:**

Plasma EV-derived S100A9 could be useful in personalising therapy in patients with CRCLM.

## Introduction

Colorectal cancer (CRC) is the 3rd most common type of cancer with high motility rate [1]. Current treatment regimens involve chemotherapy and anti-angiogenic agents (anti-VEGF, Bevacizumab: Bev), however, over 90% of these patients will succumb to their disease in less than 5 years [2].

CRCLM is intrinsically complex and heterogenous occurring primarily as at least two major histological subtypes or growth patterns (HGPs) [2–5]: desmoplastic HGP (dHGP) and replacement HGP (rHGP). Desmoplastic lesions have a stromal cell layer that separates the cancer cells from the liver parenchyma and derive their blood through angiogenesis while replacement lesions, cancer cells infiltrate the liver parenchyma, co-opts with pre-existing vasculature hence, known as vessel co-option [2, 4]. Neoadjuvant Bev+ chemotherapy had shown to be more effective in dHGP than rHGP in CRCLM patient [2]. Anti-angiogenic inhibitors could have negative outcome in rHGP lesions and properly identifying responders (desmoplastic) and non-responders (replacement) could personalised anti-angiogenic agents use [2], but current non-invasive methods are unreliable in distinguishing between the two HGPs in a clinical setting.

Biomarkers in the circulation (liquid biopsy) have emerged as a powerful tool in guiding clinical decisions. Extracellular vesicles (EVs) are a particularly interesting analyte as they contain various informative cargoes such as protein, RNA, metabolites etc. providing fingerprints of EV secreting cells [6]. EVs have been implicated as mechanistic components of the metastatic process including LM [7–9]. Most studies have focused on cancer cells, and studies from Coffy’s lab and Park’s lab looked at plasma-derived EVs in CRC patients, with a focus on proteins identified multimarket combinations that had a 98% accuracy in diagnosing CRC. Interestingly, a subset of markers, EGFR, EpCAM and GPA33 overlapped between the studies [10]. EVs may also alter the composition and metabolic state of cells within the TME, thus impacting the signature from the TME. Recent studies have investigated the impact of macrophage-derived EVs on CRC cells, identifying distinct miRNAs that increase migration, invasion, and colony formation [11]. Despite these developments, the EV-related protein signatures of HGPs associated with CRCLM have not been identified [12, 13].

We reasoned that different cellular compositions of dHGP and rHGP could be expected to result in different repertoires of EVs shed by these lesions into the circulation. Therefore, we sought to identify blood-based EV biomarkers to distinguish patient with dHGP vs rHGP lesions, enabling better stratification. We isolated EVs from plasma of CRCLM patients and analysed them for differentially expressed proteins. We identified a panel of EV-associated proteins that could successfully differentiate between desmoplastic and replacement lesions, with S100A9 exhibiting the highest diagnostic association. Interestingly, while cancer biomarkers are often sought exclusively among cancer cell proteins, S100A9 was traced back to components of the tumour microenvironment (TME), specifically in macrophages and neutrophils at the tumour-liver interface in replacement lesions. Our study demonstrated that EV-associated S100A9 can identify CRCLM subtypes, with significant therapeutic implications. Importantly, our study shows that soluble S100A9 in plasma is not diagnostic, highlighting the importance of EV-associated S100A9 in this setting.

## Materials and methods

### Human clinical sample collection and isolation of EVs using differential centrifugation method

The study was conducted in accordance with McGill University Health Centre (MUHC) Institutional Review Board (IRB) guidelines. Plasma and tissues were collected through the MUHC Liver Disease Biobank, (LDB: MUHC). Patient inclusion criteria: confirmed CRCLM diagnosis, chemonaive before and after resection of the metastatic liver tumour. Prior written informed consent was obtained from all subjects to participate in the study (protocol: SDR-11-066). Resections were performed between November 2018 and October 2020. The clinical data was collected through hospital database and medical records. Included within the data are demographics, primary and metastatic disease characteristics, relevant laboratory results (CEA, liver enzymes: ALT, AST) and comorbidities. The median age of diagnosis was 63 (range 31–81) years. Rectal carcinoma accounted for 34% of the cases. Approximately two-thirds (64%) of the patients had synchronous LM (developed metastasis within a year of diagnosing the primary tumour).

We identified 20 chemonaive patients that underwent surgical resection. Hematoxylin and eosin (H&E) stained sections were prepared for all cases for an initial histopathological assessment. The growth patterns of each section were determined by pathologists (ZG) using the consensus histopathology scoring guidelines [5].

Blood samples from 13 chemonaive patients were only available, due to volume constraints (7 dHGP and 6 rHGP lesions). Bloods were collected at two time points: (1) With disease, prior to resection and (2) 3–4 weeks after resection. Plasma was isolated by centrifuging 10 ml EDTA blood samples twice at 2500 x *g* at RT, for 15 min [14]. EVs were isolated, first by high-speed centrifugation at 10,000  x *g* for 25 min, the supernatant was then collected and prefiltered (0.22um filter: Ultident Scientific; catalogue 229747), transferred to a clean ultracentrifuge (UC) tube and centrifuged at 110,000 *g* for 70 min. The  pellet was washed using Dulbecco modified phosphate buffered saline (DPBS) and re-ultracentrifuged. The final pellet was reconstituted in 300 μl of cold DPBS and stored at −80 °C for future use.

### Nanoparticle tracking analysis (NTA)

Malvern NanoSight NS300 instrument and NTA 3.1 Software were used to determine EV particle size and concentration. EV samples were diluted in DPBS to achieve a concentration of 10^6^ to 10^9^ particles/mL, 3 videos were recorded with camera level 15 and detection threshold of 5 and blur size on auto [15].

### Protein quantification

Protein estimation was performed based on manufacture’s recommendation microBCA ^TM^ Protein Assay, (Thermo Fisher Scientific (TFS): Catalogue 23235) and ran on Tecan microplate reader (Tecan Infinite 200 Pro) [15].

### Western blot

Western blot was performed as previously described [15]. Incubated overnight at 4 °C with primary antibodies against CD81 (catalogue ab79559: Dilution 1:1000) and syntenin (abcam, catalogue ab133267: dilution 1:1000), followed by incubation with a secondary rabbit-IgG-HRP antibody (Bio-Rad: Catalogue 170–6515 dilution 1:10,000) for 1 h. Bands were detected using the ImageQuant LAS4000 imager [15].

### Transmission electron microscopy

TEM was performed as previously described [15]. Briefly, Isolated EVs were fixed with 2.5% glutaraldehyde fixative solution and then dropped onto a TEM copper grid, and incubated for 20 min, washed, and blocked with 2% bovine serum albumin 2% Casein/0.5% ovalbumin (BCO). Samples were negatively stained with 4% uranyl acetate. Images were obtained using FEI Tecnai *G*^2^ Spirit Transmission electron microscopy [15].

### Mass spectrometry proteomics

#### In-solution digestion

In-solution digestion method was previously described in detail [15].

#### Proteomics data processing and analysis

The raw data MS files were processed for LFQ analysis using MaxQuant 1.6.17.0 as per Tyanova et al. [16]. For MS/MS spectra, the Andromeda search engine was used against the Homo *sapiens* Uniprot and ensemble proteome database (GRCh38). Parameters used for processing the data includes Trypsin digestion, maximum of 2 missed cleavages, fixed modification was selected as Oxidation (M), while variable modification was selected as Acetyl (Protein N-term), and Carbamidomethyl (C), Mass tolerance set to 4.5 (MS1) and 20ppm (MS2) and peptides for quantification as unique + razor. PSM and protein FDR threshold were selected as 0.01 [16].

### Gene-set enrichment analysis (GSEA)

We conducted a GSEA using GSEA_4.2.3 software. Our input dataset comprised the entire proteome data. The analysis was performed on Molecular Signature Database (MSigDB) version 7.5.1, specifically utilising the C5 Ontology genesets collection. This collection includes genesets from both the Gene Ontology (GO) and Human Phenotype Ontology. Within the C5 Ontology genesets collection, we focused on three distinct components: Biological Process, Cellular Component, and Molecular Function. These components provide comprehensive coverage of genesets associated with specific aspects of cellular activities and functions. The GSEA analysis was executed with default parameters set by the GSEA_4.2.3 software. These default parameters encompass statistical methods, normalisation techniques, and scoring algorithms employed to identify and evaluate significantly enriched genesets.

### Perseus and processed data analysis

We analysed the MaxQuant output data (S1. Excel), using Perseus analysis tool 1.6.14.0 described in Tranova et al. [17] The LFQ output loaded into Perseus, filtered based on categorical rows, removing ‘matching rows’, ‘potential contaminants’, and ‘reverse’. We only included LFQ values detected in at least 50% of the samples, removing anything less as not significant. We tested the analysis by replacing the missing values with normal distribution and constant value. We used a two-sample *t*-test to compare between groups and plotted the differentially expressed genes in Volcano plot and PCA. For replacing the missing value, using the constant value method, we observed clear segregation of dHGP and rHGP. The tumour size information was obtained from the clinical reports, which adhere to the guidelines set by the pathologist. We primarily employed the tumour size data for the purpose of normalising our proteomics data. Normalising the data based on tumour size did not affect the results, therefore, we analysed the data without tumour size normalisation to avoid further loss of potential signatures.

We used FunRich tool for characterisation of the EV protein using EV proteomics databank i.e., Vesiclepedia and for the cellular enrichment analysis [15].

### ExoView

We processed 2 ml plasma samples (*n* = 3)/cell culture sample (*n* = 3) with qEVoriginal/70 nm Gen2 Izon column ICO-70 as per manufacturer’s instructions. We used ExoView human tetraspanin chips with cargo (Unchained Lab Catalogue 251-1029) to capture CD81, CD9, CD63 with mouse IgG control. Included anti-syntenin AF555 at 1:200 dilution as an intra-EV marker and anti-S100A9 APC at 1:60 dilution (Catalogue BL350708). The chip was then scanned with ExoView (R200).

### Immunohistochemistry and scoring

We performed IHC on FFPE tissue sections using the standard protocol [18]. The antigen unmasking step was performed by following the company’s recommendation. Briefly, slides were Incubated overnight at 4 °C with primary antibody anti-S100A9 (TFS, Catalogue PA5-82145, dilution 1:8000), anti-CD4 (R and D systems, Catalogue AF-379-NA, dilution 1:1200), anti-CD8 (Cell signalling, Catalogue 70306, dilution 1:200), anti-CD68 (Catalogue ab201340, dilution 1:4500) and Elastase (R&D Systems, Catalogue MAB91671, dilution 1:1000) and used secondary HRP Anti-Rabbit and mouse (Dako K400311-2 and K4003111-2). All slides were scanned at 40x magnification using the Aperio AT Turbo system.

### Immunofluorescence (IF)

We performed IF on FFPE tissue sections (*n* = 3 rHGP samples) and captured 3 images of distinct regions (distal liver, interface, and tumour region) for each slide. Heat induced epitope retrieval was performed using either using sodium citrate (pH 6) or Tris/EDTA (pH 9) in a steamer. The sections were incubated with primary antibodies against: CD68, Elastase and conjugated antibodies against: Arg1 APC (Catalogue BL369706, dilution 1:50), iNOS PE (Novus Bio, Catalogue NBP2-22119PE, dilution 1:50) and S100A9- APC (BL350708, dilution 1:400). The sections were then incubated with appropriate secondary antibodies i.e., AlexaFluor 488 goat anti-mouse IgG (1:1000, Invitrogen cat. 10860) for 2 h at RT followed by incubation with DAPI (1:1000; TFS) for 10 min. The slides were visualised using Zeiss confocal microscope system [18].

### Colocalization analysis

To generate the colocalization overlays and determine the percentage of colocalized pixels, we utilised the colocalization Finder plugin within the ImageJ software. The plugin generates scatter plot with intensity of the *x* and *y*-axis signal representing each positive signal (S100A9 and CD68/Elastase). To set the threshold for the positive signal we used distal liver control and set the intensity value accordingly. Pixels that exhibited positive signals in both channels were considered as co-localised [18].

### Immune cells isolation and flow cytometry

In all, 20 ml of whole-blood patient sample (*n* = 3) was collected with an EDTA tube. PBMC isolation was performed using Easysep™ Direct human PBMC isolation kit Cat 19654 with EasySep magnet Cat#18001 from stem cell following the manufacture’s protocol. The immune cells are cultured using the method described by Kwak et al. [19] by treating with GM-CSF. On the seventh day the media was changed with 8 ml of RPMI 1640 media, incubated and the condition media was collected after 78 h.

For the characterisation of the human MDSC, we followed the method described by Bao et al. and Lee et al. paper for selection of the markers [20, 21]. We used following antibodies: anti-CD45 BUV395 antibody (BD Biosciences catalogue 563792), anti-CD11b BV650 antibody (catalogue BL 101259), anti-CD14 PerCP antibody (BD Biosciences catalogue 340585), anti-CD15 PE antibody (BD Biosciences catalogue 555402), anti-HLA-DR PE-Cy7 antibody (catalogue BL307616), anti-CD68 BV711 antibody (BD Biosciences catalogue 565594), anti-CD86 BV421 antibody (BD Biosciences catalogue 566454), anti-CD206 FITC antibody (BD Biosciences catalogue 551135), and anti-S100A9 APC antibody (catalogue BL350708), and Viability Dye eFluor 780 (TFS Catalogue 65-0865-14). For voltage gating, we used compensation beads (UltraComp eBeads™ Compensation Beads, TFS, Cat 01-2222-42) and we also ran Fluorescence minus one (FMO) control for all the marker to establish negative gating. For the immune cell fixation and permeabilization, we used Transcription factor buffer set (BD Biosciences Cat#562574) and Stain buffer FBS (BD Bioscience 554656) for washing cells and eluted immune cells with 300 μl of stain buffer to take reading on BD LSRFortessa flow cytometer.

### Flow cytometry data analysis

We used Flowjo_v10.8.1 for analysing our data to characterise S100A9 secreting immune cells. A common template was set for gating the immune cells markers and characterised the immune cells. Further, we concatenated the data (*n* = 3) and performed analysis on the same.

### S100A9 ELISA

Direct plasma (100 μl) or isolated EVs from 4 ml plasma using UC method. The EV pellet was eluted with 300 μl of D-PBS and lysed with RIPA and anti-S100A9 ELISA was performed in triplicates. Human S100A9 DuoSet ELISA (DY5578) and DuoSet ELISA Ancillary Reagent Kit 2 (DY008B) from R&D Systems were used following manufacturer’s recommendations.

### Statistics

The comparative analysis between Desmoplastic and Replacement HGPs in both proteomics and IHC was performed using T-sample test with mean, standard deviation, and *p*-value < 0.05. GraphPad Prism 9 was used for the IHC data analysis, while Perseus statistical tool for the proteomics data analysis. ROC-AUC was also performed with standard error, 95% confidence interval and *p*-value < 0.05.

## Results

### Identification of liver metastasis subtypes in patients with disseminated colorectal cancer

**Fig. 1:**
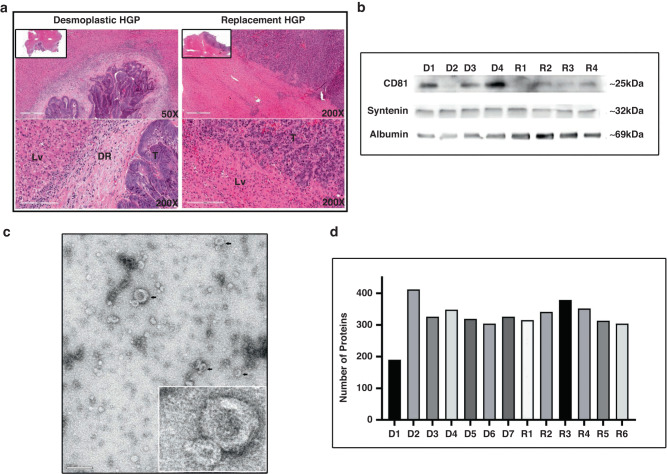
Characterisation of extracellular vesicles: **a** H and E stain showing both dHGP and rHGP of CRCLM. **b** Western blot with CD81 and Syntenin as EV markers. **c** Visualisation of EV with TEM (scale bar 200 nm). **d** Distribution of number of proteins in each sample. D desmoplastic samples, R replacement samples.

Recent studies identified two major subtypes of LM associated with CRC: desmoplastic (angiogenic) and replacement (non-angiogenic) HGPs, with dramatically different responses to the standard of care [2]. We asked whether these different forms of metastatic disease can be reflected in circulating EVs as potential liquid biopsy analytes. To identify patients with the preponderance of the respective forms of liver metastatic growth, H&E staining was performed on CRCLM tissues included in the study and scoring of the HGPs as desmoplastic (dHGP) or replacement (rHGP), or their co-existence was performed by a licensed pathologist (ZG). Since our previous analysis indicated that patient lesion that scores above 50% for desmoplastic HGP predicts significantly better survival rates under chemo-anti-angiogenic adjuvant therapy [2], we followed similar cut-offs in our present study comprising 13 chemonaive patients 7 with predominantly dHGP and 6 with rHGP (S2 Table 1). It should be noted that LM (Fig. 1a) are complex ecosystems containing cancer cells, stroma, vasculature, and inflammatory infiltrates each of which can be potentially a source of liquid biopsy signals in blood.

### Characterisation of EV from plasma sample

EVs offer a unique platform of liquid biopsy diagnostics given their considerable numbers in blood as well as intrinsic complexity that offers a multitude of molecular signals reflective of their parental cells. EV isolates from 11 chemonaive CRCLM patients were characterised based on MISEV2018 [22]. We performed NTA in all the samples to show the particle distribution (S3 Fig. A). The particle sizes detected by NTA ranged from ~116–135 nm in dHGP and ~107–144 nm in rHGP, with no significant differences observed in average total particle concentration (S3 Fig. B, C). Isolated EVs from patients with rHGP after resection (3–5 weeks after resection) showed significant 3.2-fold increased average total number of the particle (*p*-value 0.026) compared to rHGP, but this is not same in case of the resected dHGP (S3 Fig. B). CD81 (EV membrane marker) and syntenin (intra-EV marker) were detected in all our EV samples using Western blotting and Nano View imaging (Fig. 1b). TEM image of EV revealed the presence of heterogenous particles including cup-shaped structures 100–200 nm in size and morphology compatible with EVs (Fig. 1c). Overall, plasma isolates were enriched for mostly small, exosome-like EVs and similar characteristics across the patient cohort.

### Proteome of plasma-derived EVs from colorectal cancer patients

We analysed the proteome of circulating EVs from patients using LC-MS/MS platform. A median of 326 proteins per EV samples were detected, with relatively uniform distribution of proteins across all samples, except for one sample (Fig. 1d). These yields are in keeping with the average EV protein content reported in the literature [7]. Most of the EV-associated proteins identified overlapped with protein found in publicly available EV proteomics database i.e., Vesiclepedia (63%) and ExoCarta (Top 100 EV proteins) (84.01%) (S4 Fig. A). We additionally identified 97 unique EV proteins not found in Vesiclepedia. Cellular component enrichment analysis showed highest percentage of the protein detected were of exosome origin (67.5%) (S4 Fig. B). Comparison of proteomic data in the presence of disease vs after surgical resection showed high similarity, with 93% and 90% similarity in patients with desmoplastic lesions and replacement lesions, respectively (S4 Fig. C). The similarities were also observed when cellular enrichment analysis was performed (S4 Fig. D).

### EV-derived proteome differentiates between subtypes of liver metastasis in colorectal cancer patients

While the global proteomic profiles of plasma-derived EVs was similar across the patient cohort a more detailed analysis revealed several intriguing differences. Using GSEA, 61 genesets were significantly enriched (*p*-value < 0.005) in rHGP patients, associated with cell proliferation and migration (S5 Fig. A, C). This is of interest as we have previously shown migration as an important characteristic of rHGP lesions [2, 23], relative to their dHGP counterparts. In other words, proteomes of circulating EVs from cancer patients reflected some of the characteristics of metastatic tumour tissues of these patients. The dHGP proteomics data only 6 genesets significantly enriched (S5 Fig. B) associated with adaptive immune response (S5 Fig. D) and B-cell activation (S5 Fig. E).

**Fig. 2:**
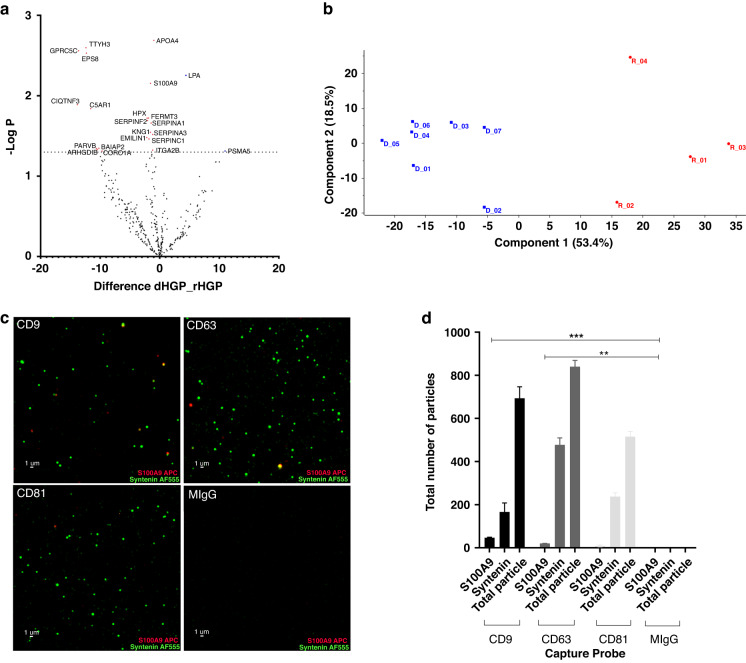
Proteomics analysis performed between desmoplastic (*n*  = 7) and replacement (*n* = 4). **a** Volcano plot showing 22 differentially expressed significant proteins. **b** Unsupervised PCA performed using differentially expressed significant proteins. **c** ExoView data showing S100A9 (Red) and Syntenin (Green) image from plasma EV (*n* = 3). **d** ExoView graph representing S100A9 signal with different capture probe.

On our initial analysis using unsupervised principal component analysis (PCA) we observed two rHGP samples as outliers, segregating close to the dHGP lesions and not the rHGP lesions. Upon further analysis these lesions had a histological growth pattern of 50% dHGP and 50% rHGP. To identify a more robust marker we omitted these two samples from further downstream analysis. Interestingly, these two samples had an overall survival similar to dHGP lesions (data not shown) and thus behaved more like dHGP patients. Two-sample *t*-test identified 22 differentially expressed proteins between the two HGPs (7 dHGP and 4 rHGP) (Fig. 2a), which were subjected to unsupervised principal component analysis (PCA) and ROC analysis. The unsupervised PCA now showed clear clustering of the two HGPs, suggesting their distinctive molecular profiles and potential use for diagnostic stratification (Fig. 2b). Fifteen out of 22 proteins had an AUC cut-off >0.8 and 6 of these proteins have been reported in the literature to correlate with cancer and the TME (S6 Table 2). These results suggest the potential use of EV-associated proteome for diagnostic stratification of HGPs.

### EV-associated S100A9 expression correlates with the replacement subtype of liver metastasis

Two EV-associated proteins, S100A9 and APOA4 were found to differentiate between HGP lesion subtypes with a high degree of accuracy (S6 Table 2). While this score could be affected by the small sample size this observation is intriguing for at least two reasons. First, we observed a close correlation of EV-associated S100A9 with rHGP, but not dHGP type lesions, which encourages further studies of its biomarker potential. Second, S100A9 is known to be expressed by myeloid-derived suppressor cells (MDSC) and promotes polarisation of M2 macrophages present in the TME of many cancers including CRC. The increased expression of S100A9 has also been shown to be associated with CRC staging, metastasis, and a potential diagnostic marker [24–26]. ExoView, a chip-based ELISA, confirmed the presence of S100A9 in plasma of CRCLM EVs. (Fig. 2c, d). However, S100A9 containing EVs were found to be relatively scarce, comprising approximately 6.8% and 2.4% of the EV population in CD9 and CD63 positive samples, respectively.

### Expression of S100A9 colocalizes with myeloid cells infiltrating replacement-type liver metastases

**Fig. 3:**
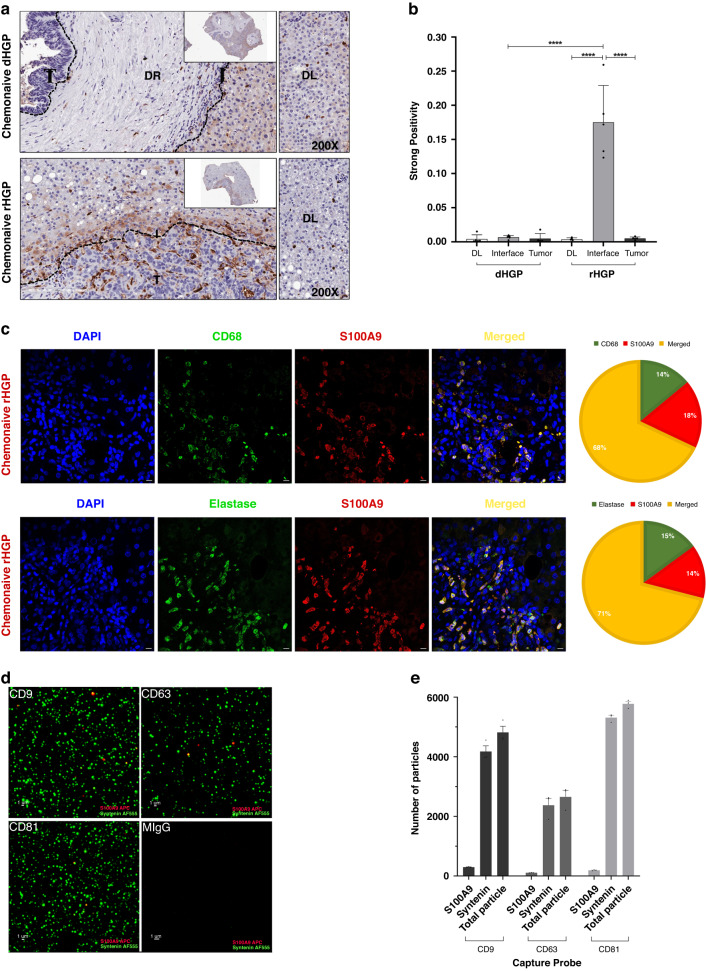
Expression of the S100A9 in TME. **a** IHC showing increased S100A9 expression in replacement lesions of Chemonaive CRCLM at the Interphase region (*n* = 5). **b** Quantitation of different region of CRCLM S100A9 expression in both Desmoplastic and Replacement HGPs. **c** Representative images of colocalization of macrophage (CD68) and Neutrophil (Elastase) and S100A9 at the interphase of Desmoplastic and Replacement Chemonaive CRCLM tissue with elastase and S100A9 colocalization score of 0.842 and CD68 and S100A9 of 0.825 (*n* = 3 rHGP FFPE tissue slides). **d** ExoView data showing S100A9 (Red) and Syntenin (Green) image from GM-CSF treated PBMC condition media (*n* = 3). **e** Quantitation of the S100A9 and syntenin expression.

While EVs-isolated tumour markers are often sought among molecules expressed by cancer cells, we chose to explore whether this is the case for S100A9 protein present in EVs isolated from plasma of CRC patients with rHGP-type of liver metastasis. To this end we stained for S100A9 expression and found similar expression in the liver parenchyma associated with both lesion types, especially within the sinusoids, suggesting the staining of Kupffer cells. Interestingly, we observed a significantly higher level of S100A9 expression at the interface of the replacement lesions compared to other areas of the samples and significantly higher levels compared to desmoplastic lesions (Fig. 3a, b).

Since S100A9 has been shown to be expressed in myeloid cells (MDSCs and macrophages and neutrophils) [20, 27, 28], we investigated whether this holds true for rHGP. Interestingly, we found this to be the case as an enriched population of neutrophils and macrophages expressed an abundant S100A9 positive signal, which correlated with the respective markers (CD68 and Elastase) with Pearson correlation *r*-values of 0.842 (neutrophils) and 0.825 (macrophages) (Fig. 3c). These values were markedly higher than in the case of corresponding cells in desmoplastic lesions with 0.576 and 0.544 respectively (Fig. 3c), which were scarcely present in these tumours.

### Myeloid cells release EVs containing S100A9 in vitro

The study found that macrophage express S100A9 within the rHGP microenvironment, but it is unclear whether they secrete EVs containing S100A9 or contribute to the EV-associated S100A9 signal in plasma. To investigate this, PBMCs were isolated, cultured in vitro with GM-CSF, flow cytometry confirmed the presence of S100A9 in MDSCs and classical monocytes (S6 Fig. A). We used GM-CSF not only to differentiate the MDSCs, but also to increase the expression of S100A9 as it is required for M1 and M2 macrophage polarisation (S6 Fig. B) [19]. EVs were isolated from the conditioned media and analysed using ExoView platform, revealing that S100A9 is present in a subset of MDSC EVs often co-expressed with CD63, CD81 and CD9 with percentage distribution of 4%, 3.3% and 6% respectively (Fig. 3d, e). These results suggest that MDSCs and classical monocytes may be a potential source of EV-associated S100A9, contributing to its presence in circulation.

### Replacement-type liver metastases are replete with immunosuppressive cells

Our observations suggest that EV-associated S100A9 protein in the circulation correlates with elevated expression of this protein in myeloid cells present within rHGP-type (but not dHGP type) LM. To gain further insight into the significance of this connection we analysed immune profiles of rHGP and dHGP metastases including both the innate and adaptive immune landscapes.

**Fig. 4:**
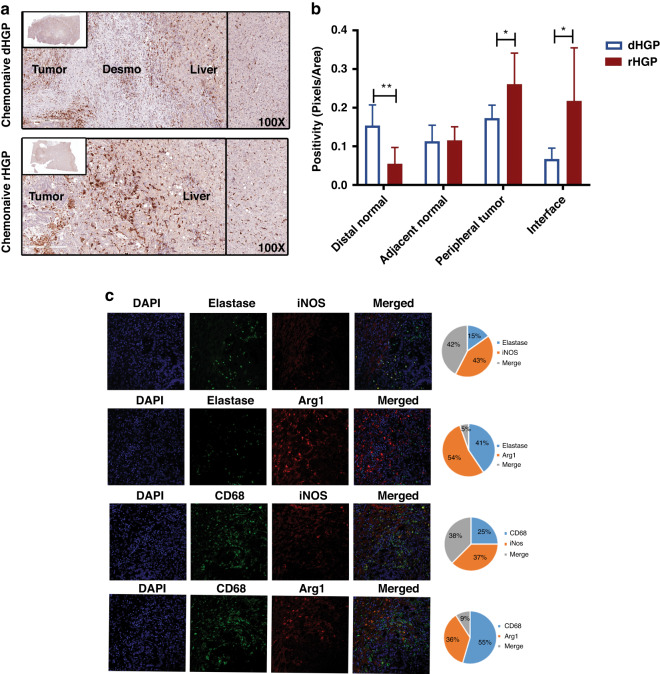
Abundance of innate immune cells in rHGP. **a** Anti-CD68 IHC for Desmoplastic and Replacement CRCLM. **b** Quantitation of the anti-CD68 at multiple regions in CRCLM. **c** Immunofluorescence showing the colocalization of the Macrophage (CD68) with Arg1 and iNOS.

First, we stained rHGP and dHGP lesions for markers of macrophages (CD68) (Fig. 4a). In the dHGP metastases the liver parenchyma demonstrated scarce CD68+ cells, which were similar in numbers of the adjacent normal and interface regions. There was a significant decrease in CD68 positivity in the peripheral tumour regions with areas lacking infiltration (Fig. 4b) In contrast to dHGP, rHGP lesions demonstrated ample CD68+ cells the concentration of which was apparent at the interface and within the adjacent tumour compared to distal liver parenchyma (Fig. 4b). Notably, the greater presence of CD68+ cells in rHGP lesions correlated with the expression of S100A9.

Since the expression of S100A9 was reported to promote polarisation of macrophages to M1 and M2 phenotype in many cancer types [19]. Therefore, we explored the activation state of both macrophages and neutrophils in rHGP vs dHGP metastatic lesions. We adopted established criteria for the macrophage M1 phenotype (CD68^+^, iNOS^+^, Arginase1^-^) vs. M2 phenotype (CD68^+^, iNOS^-^, Arginase1^+^) (Fig. 4c) [21]. Staining for these markers revealed that dHGP metastases had very few macrophages (Fig. 4a). On the other hand, iNOS and Arginase1 were expressed by macrophages in rHGP lesions especially at the liver–tumour interface, suggesting the presence of both M1 and M2 cells (Fig. 4a). Macrophages in the normal liver parenchyma were negative for both markers.

**Fig. 5:**
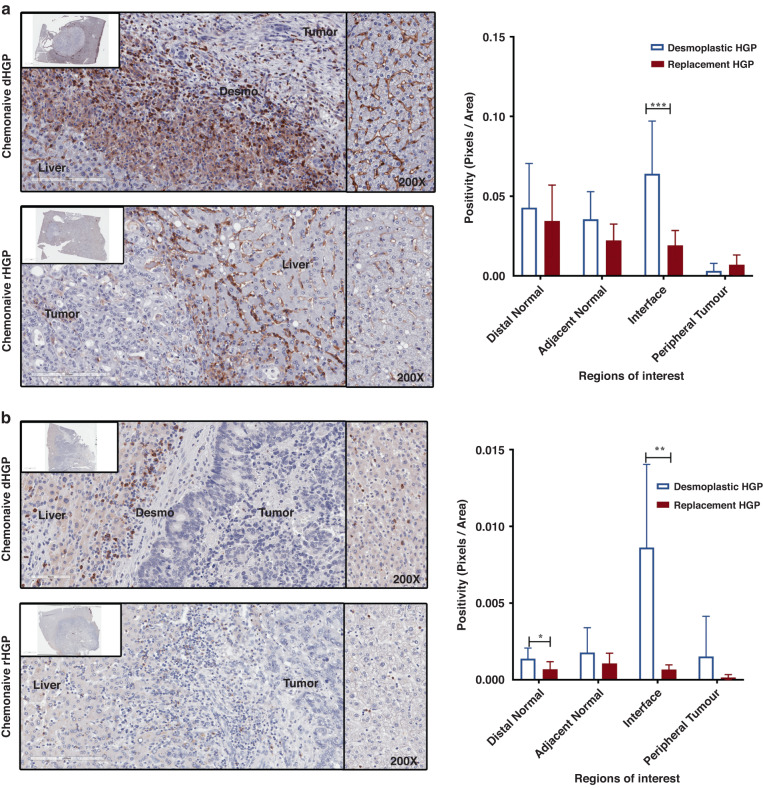
Increased T cell in TME of desmoplastic HGP. **a** Increased CD4 positive cell expression in interface region of the Desmoplastic Lesion. **b** Increased CD8 positive cell expression in interface region of the desmoplastic lesion.

It has also been shown that S100A9 expressing MDSCs can suppress T cell response in cancer and other diseases [29]. We then stained serial sections of 11 chemonaive desmoplastic and 9 replacement lesions for T helper cells (CD4^+^) (Fig. 5a) and cytotoxic T cells (CD8^+^) (Fig. 5b). rHGP lesions revealed a barely detectable signal for both CD4 and CD8 cells throughout the lesions, conversely, we observed an increased number of both immune cell types at the tumour-liver interface of dHGP lesions. This staining dramatically decreased towards the centre of the tumour. Digital quantification of CD4 and CD8 (Fig. 5a, b) confirmed that there was a significantly higher amount of CD4 and CD8 at the interface of dHGP lesions compared to the background liver parenchyma. To further define the subtype of T cells, present at the dHGP interface we stained these tissues for markers of regulatory T cells (Treg) such as FoxP3. We observed a significantly higher staining of Tregs (FoxP3^+^ cells at the interface of dHGP lesions, suggesting the existence of an immunosuppressive environment. T cells in the normal liver parenchyma distal to the tumour were generally not found to express FoxP3. Our visual observations of the number of CD4^+^ relative to CD8^+^ cells in dHGP lesions demonstrated a larger percentage of CD4^+^ cells at the interface, adjacent normal and in pockets of inflammation present in the liver parenchyma (Fig. 5). Thus, immunophenotypes of dHGP and rHGP lesions are markedly different, and the molecular signal corresponding to the latter lesions (S100A9) is readily detectable in plasma EV of patients.

### Preferential release of S100A9 protein into circulating plasma as cargo of extracellular vesicles

**Fig. 6:**
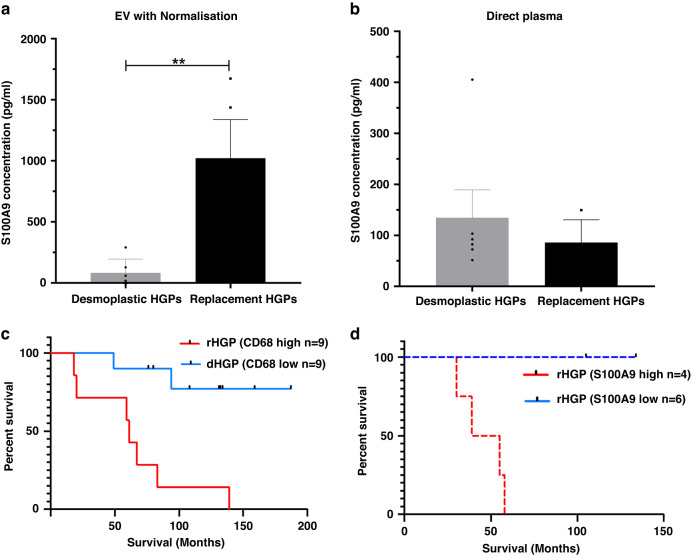
S100A9 and CD68 expression associated with the patient survival: **a** ELISA showing significant increased expression of plasma-derived EV S100A9 (Chemonaive dHGP *n* = 6, rHGP *n* = 4). **b** ELISA showing significant increased expression of plasma S100A9 (Chemonaive dHGP *n* = 6, rHGP *n* = 4). **c** Comparative survival analysis of CD68 signal in tissue of dHGP and rHGP. **d** Comparative survival analysis of plasma-derived EV S100A9 expression in desmoplastic and replacement lesions.

We next asked whether the isolation of EVs is required to observe enrichment of S100A9 protein in plasma of patients with rHGP metastatic lesions. To address this question, we used an ELISA assay to screen both total plasma samples and EVs isolated from plasma for the levels of S100A9 protein. As expected, we observed increased expression of S100A9 in plasma EV isolates from patients with rHGP lesions relative to their dHGP counterparts (Fig. 6a). Surprisingly, analysis of total plasma S100A9 revealed no such difference (Fig. 6b).

### Macrophage infiltration and plasma levels of EV-S100A9 correlate with patients survival

Overall, our data suggest that enrichment of plasma EVs in S100A9 correlates (and may be driven by) the macrophages and neutrophils present in rHGP tissue. Since rHGP-type of LM predicts poorer response to current standard of care and survival we reasoned that the nexus between myeloid cell infiltration and elevated plasma EV-S100A9 may correlate with clinical outcomes. To further investigate this question, we examined the levels of CD68+ cells in LM, and the corresponding levels of S100A9 in plasma-derived EVs, as a function of overall patient survival (OS). Using the positivity score with high and low cut-offs of expression, we examined whether the difference in OS exists between patients with high vs low levels of CD68. Although the sample sets were small, we observed high levels of CD68 correlated with poor OS (*p* = 0.0016) (Fig. 6c). Remarkably, high vs low levels of EV-associated S100A9 (ELISA) similarly correlated with OS (*p* = 0.049) (Fig. 6d). Thus, CRC patients with high CD68 positivity and high plasma EV-S100A9 exhibited the most unfavourable prognosis.

## Discussion

Our study brings to the fore several novel findings. First, we discovered that analysing plasma EVs reveal molecular signatures of unique biological subtypes of LM with different cellular features and mechanisms of expansion. Second, this diagnostic and predictive potential is epitomised by EV-associated S100A9 protein found at high levels in plasma of patients with predominantly rHGP-type of LM. Third, S100A9 protein’s diagnostic properties are only evident when analysed in the context of plasma EVs and not in total plasma. Fourth, our analysis suggests that host myeloid cells may be a potential source of EV-associated S100A9 protein in plasma, instead of cancer cell-specific markers typically analysed in liquid biopsy studies. Lastly, EV-S100A9 levels in plasma and tissue CD68 macrophage infiltration at rHGP metastatic tumour boundaries are strongly associated with each other and with poor patient survival.

Our study suggests that S100A9, a marker for MDSCs could potentially be used as a stratification marker for CRCLM. However, further research is necessary to understand the underlying biological mechanisms, regulation, biogenesis, and functional role of S100A9-carrying EVs. Previous literature suggests that S100A9 is involved in various functions within TME, including cancer cell migration, neutrophil recruitment, and escape from immune surveillance by cancer cells via TGFβ activation [30, 31]. Although, previous studies had reported the presence of S100A9 in EVs, there is currently no published research on the role of EV-derived S100A9 in CRCLM stratification.

Our analysis of EVs found in the circulation of patient’s blood reflects the immunophenotypes of underlying metastatic lesions. Interestingly, the adaptive pathways signal in EVs was significantly higher in dHGP lesions and not in rHGP lesions. This was then correlated to the lesion where we observed that dHGP lesions contained more lymphocytes that rHGP. Interestingly, the latter lesions were infiltrated mostly by the innate immune cells (neutrophils and macrophages) with scarce to no lymphocytic infiltration. Several papers have studied the adaptive immune cells however, except for our paper, which focused on neutrophils, the innate immune cells have not been studied. With respect to the innate immune cells, neutrophils had been previously evaluated by our group [18, 19]. We observed a considerable contribution of protumor phenotypes in rHGP lesions, indicating that these lesions have strongly immune suppressive microenvironment. This is supported by our earlier work demonstrating the expression of immunosuppressive cytokines such as TGFβ at the interface of rHGP lesions and liver parenchyma [23], where we also observed the highest density of macrophages and neutrophils. We suspect that the innate immune cells in rHGP lesions are MDSC-derived macrophages and neutrophils, supported by the higher expression of S100A9 in the TME of these lesions. Kwak et al. showed that S100A9 is required to maintain the M2 polarisation in the MDSC-derived macrophages [19], and in many solid tumours, high numbers of MDSC are associated with poor prognosis [32, 33]. Furthermore, a subset of patients treated with FOLFOX Bevacizumab chemotherapy showed accumulation of Th17 cells associated with poor prognosis, while others showed depletion of granulocytic MDSC, associated with better outcome in metastatic CRC [34]. This could be due to different lesions responding to the current treatment method, emphasising the need for biomarker-based lesion stratification. This work would also suggest that investigating the population of the immune cells expressing the S100A9 and systemic immune profiling of these two lesions will answer to some of the challenges in stratifying these two lesions and tracking the efficacy of the treatment.

One potential blood marker for rHGP is SERPINF2 (alpha-2-antiplasmin), which inhibit plasmin and is associated with poor outcome in various cancer due to its role in preventing L1CAM destruction and promoting metastatic outgrowth during brain metastasis [35]. Another candidate marker, C5AR1, is expressed in both myeloid cells and non-myeloid cells [36] and has been associated with increased recruitment and polarisation of macrophages to an M2 state in CRCLM and pancreatic cancer LM [29, 36–38]. The expression of TTYH3, another identified marker, has been associated with poor prognosis and poor response to immunotherapy in lung cancer [39] with high level of expression associated with immune cell infiltration, specifically macrophages and T-reg cells [39–48].

To improve treatment for patient with rHGP, it is important to stratify them into responders and non-responders. Our research demonstrated that this can be achieved through the use of EV-derived protein signature to distinguish between desmoplastic and replacement lesions before treatment. Additionally, EV S100A9 is a potential blood-based marker that is significantly higher in rHGP. Plasma EV-derived S100A9 reflects tissue immune status and could serve as a surrogate marker for tumour-associated macrophages. We found that patient exhibiting high levels of tissue-derived CD68 experiencing poor OS, which is reflected in circulating S100A9. Although our sample size was small, we are continuing to validate these findings in a larger patient cohort.

A limitation of this study was the number of samples. As we focused on chemonaive patient samples it is important to note that these patients are very rare. As these patients are stage 3 or 4 they are usually treated before going for surgical resection, however, we are in the unique situation to have access to these patients. Moving forward we are continuing to collect blood from these patients in clinic at diagnosis, to obtain additional chemonaïve samples. Furthermore, in this paper we are using the ultracentrifugation method for EV isolation. This method is the most common method used and allows for the identification of different sized EVs, allowing for a more robust discovery phase. One major drawback is the volume of blood required (~5–10 ml plasma) to obtain enough EVs representing our subpopulation of rare S100A9 containing EVs. Moving forward we are evaluating other methods, such as magnetic bead capture, to specifically isolate CD11b-derived EVs, thus enriching for our population. This paper was the necessary first step that identified the specific population of EVs, thus allowing us to now move to a targeted EV isolation approach.

## Supplementary information

Supplementary table

Supplementary information

## Data Availability

The proteomics data are available on the pride databank and other data are available upon request from the corresponding authors.
