# Supplementary material for: Circulating extracellular vesicles containing S100A9 reflect histopathology, immunophenotype and therapeutic responses of liver metastasis in colorectal cancer patients
Source: BJC Rep. 2023 Aug 2;1:8. doi: 10.1038/s44276-023-00007-9 (PMC11524068; doi:10.1038/s44276-023-00007-9)
Supplement: Supplementary file 2 — Supplementary information [file 44276_2023_7_MOESM2_ESM.docx]

**Supplementary**

**S1. Excel sheet with proteomics data**

|  | Desmoplastic Lesions  N=7 | | Replacement Lesions  N=6 | | T. Test |
| --- | --- | --- | --- | --- | --- |
| Histological percentage score of the Lesions | A_01 | 95% Desmoplastic, 5% Replacement | VC_01 | 100% Replacement |  |
|  | A_02 | 100% Desmoplastic | VC_02 | 100% Replacement |  |
|  | A_03 | 100% Desmoplastic | VC_03 | 2 lesions, 99% Replacement, 1% Desmoplastic |  |
|  | A_04 | Lesion 1: 100% Desmoplastic,  Lesion 2: 2% Replacement, 98% Desmoplastic | VC_04 | A: 100% Replacement, B. 80% Replacement, 20% Desmoplastic |  |
|  | A_05 | 95% Desmoplastic | VC_05 | 80% Replacement |  |
|  | A_06 | 95% Desmoplastic, 5% Replacement | VC_06 | 60% Replacement, 30% P, 10% Desmoplastic Lesion |  |
|  | A_07 | 100% Desmoplastic |  |  |  |
| Age, Q3-Q1 (IQR) | 76.5-58.5 (18) | | 65-50.5 (14.5) | | 0.16 |
| Gender | M=7 | | M=1, F=5 | |  |
| BMI, Mean (SD) | 27.47±5.67 | | 24.56±3.39 | | 0.33 |
| Location of primary tumor (1=right, 2= left/sigmoid, 3= rectum, 0=bilateral) | 0=1,1=2,2=1,3=3 | | 1=2,2=1,3=3 | |  |
| Tumor stage of primary | 1=1,2=2,3=2,4=2 | | 2=1,3=4,4=1 | |  |
| Number or liver lesions at first diagnosis | 1=6,5=1 | | 1=3,2=2,6=1 | |  |
| Diameter of largest liver lesion (cm) | 2.6±1.02 | | 2.46±1.02 | | 0.82 |
| Overall survival in months, Q3-Q1 (IQR) | 94.5-31 (63.5) | | 47.5-33 (14.5) | | 0.238638 |

**S2 Table 1. Clinical sample details**

Abbreviations: Replacement HGPs, Desmoplastic HGPs, Pushing HGPs (IQR=Interquartile range, Q3=third quartile, and Q1=First quartile)


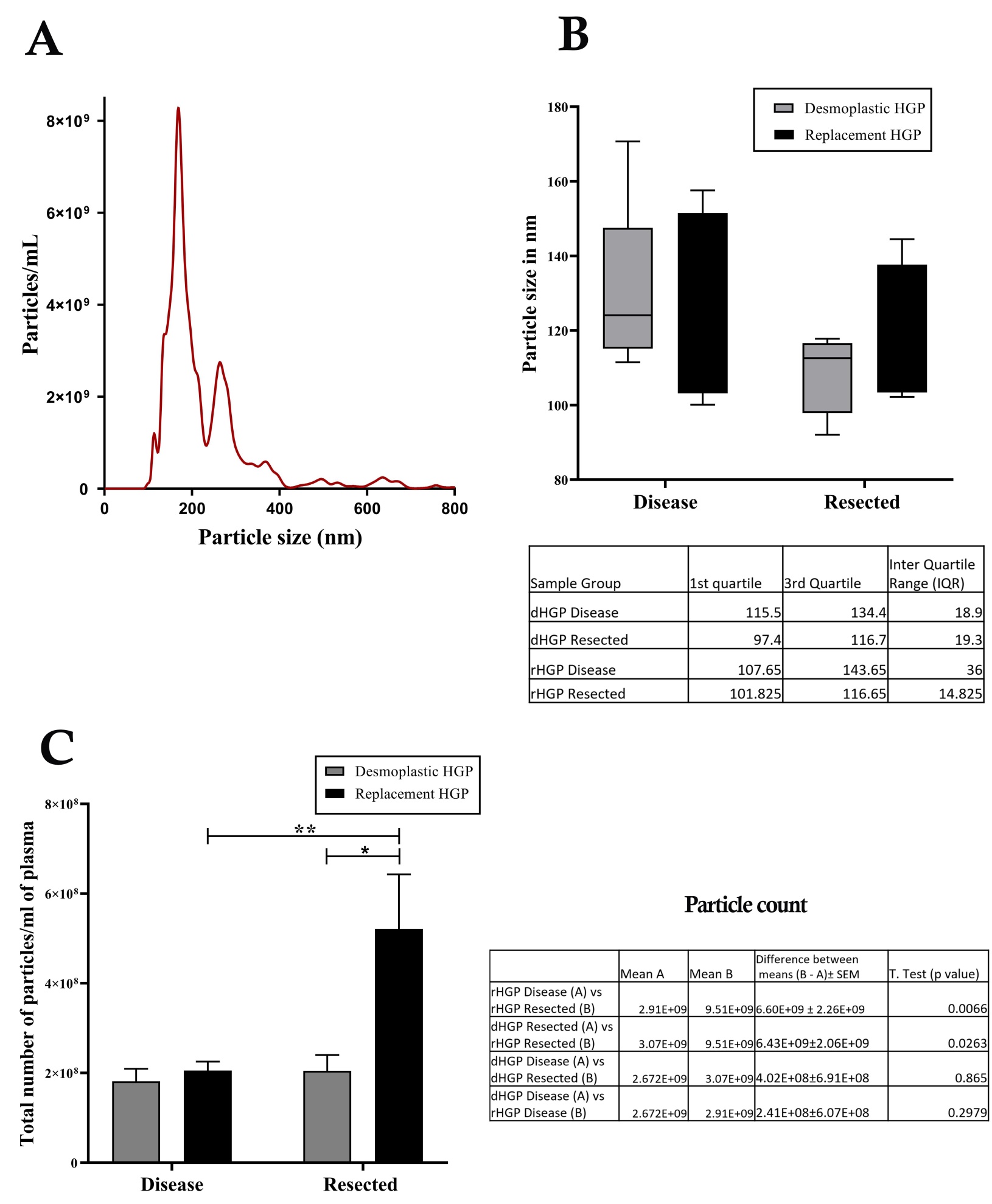


**S3 Figure: Nanoparticle tracking Analysis showing particle concentration and size in all the patient sample: A. Average particle distribution in all the samples B. Size distribution of particles detected using NTA ( dHGP n=7, rHGP n=4) C. Particle concentration in all the groups of sample (dHGP n=7, rHGP n=4)**


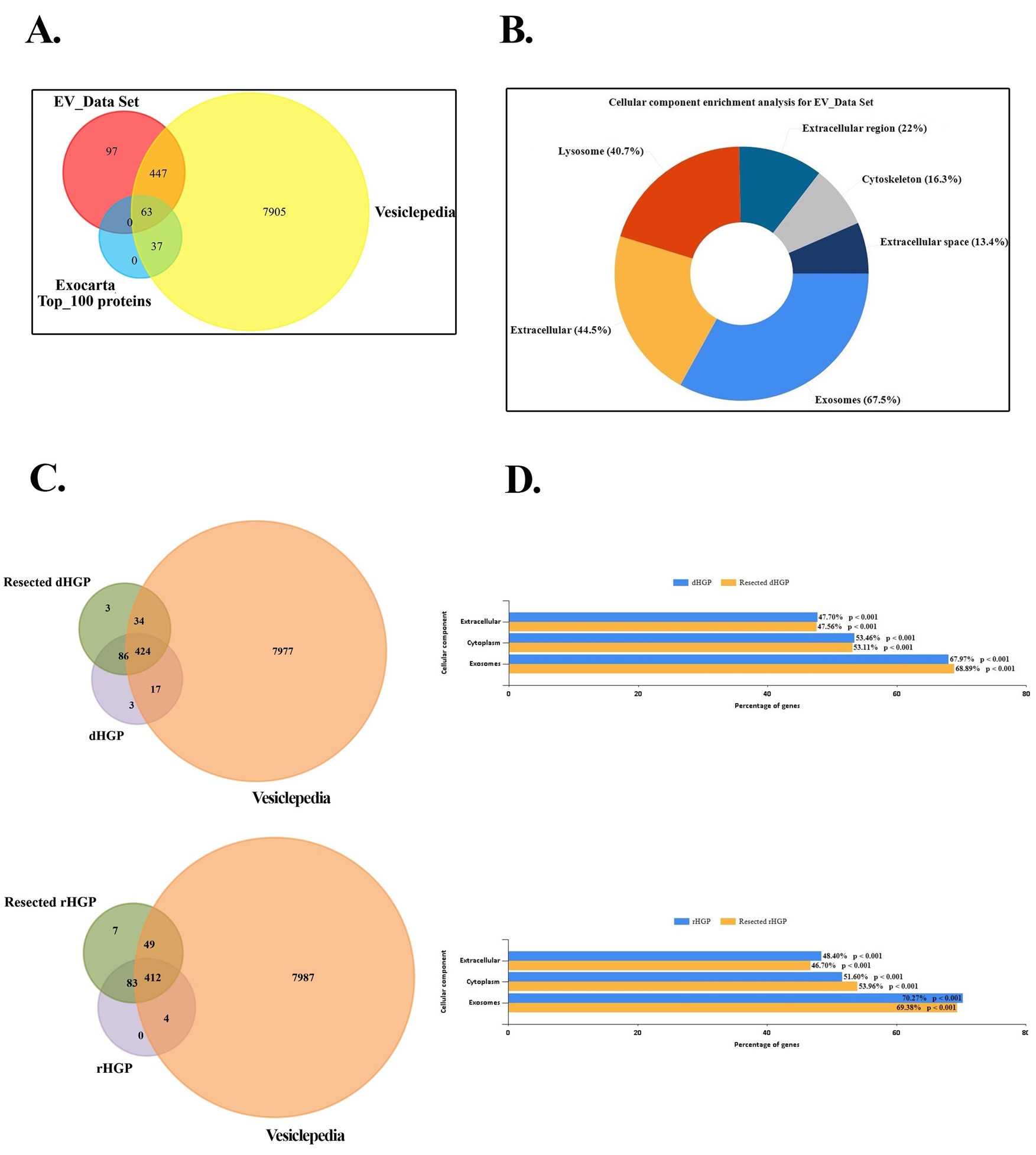
S4 **Figure: Enrichment Analysis:** A. Venn Diagram showing comparative analysis of our EV proteomic data set with Vesiclepedia and ExoCarta Top 100 proteins B. Cellular component enrichment analysis of our EV dataset C. Venn Diagram showing comparative analysis of our EV proteomic data of with disease vs resected dHGP and rHGP D. Comparative cellular enrichment analysis of our EV proteomic data of with disease vs resected dHGP and rHGP.


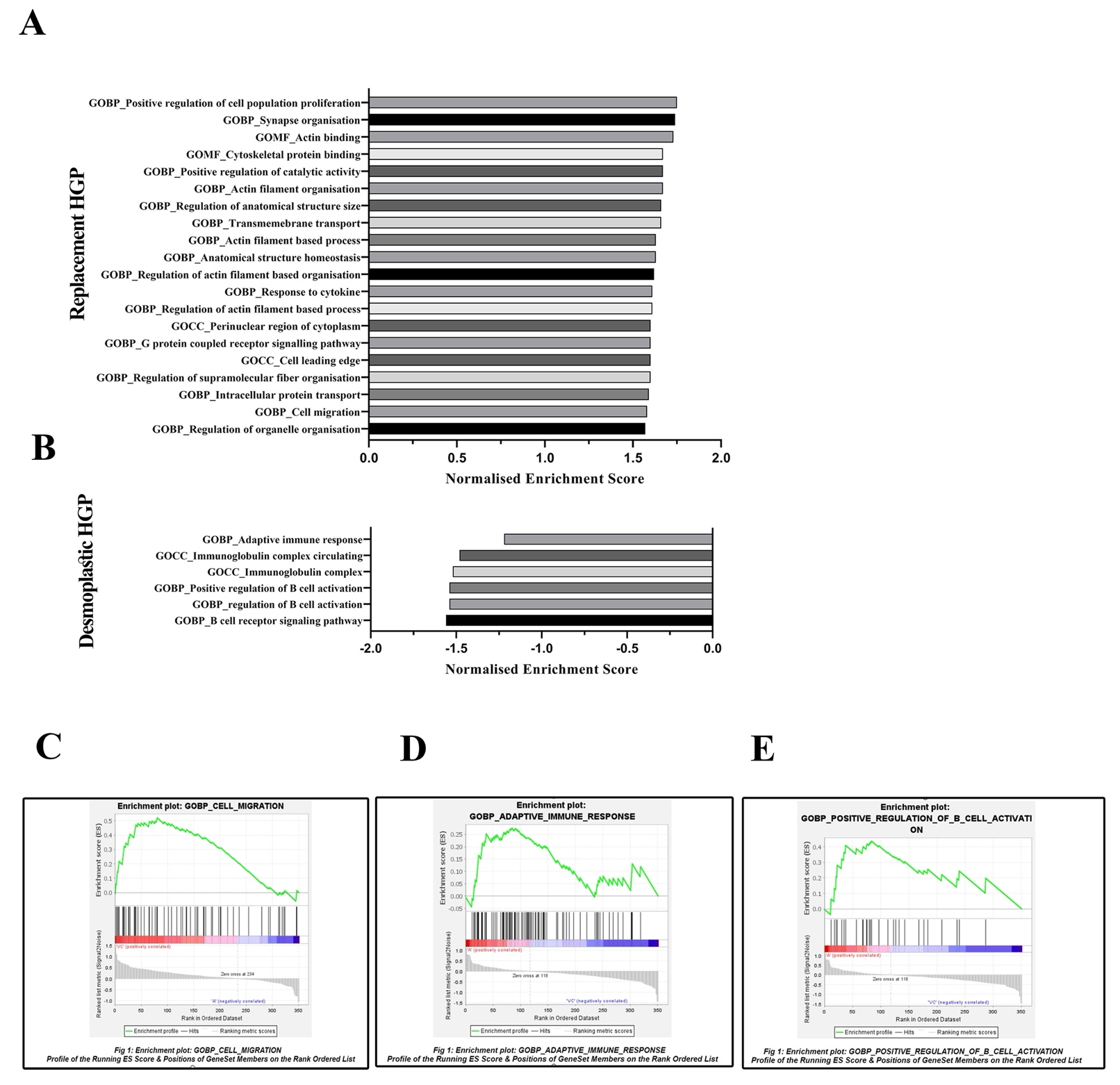


**S5 Figure: GSEA 4.2.3 with top 20 GO enrichment analysis with statistically significant p<0.05**. A. GSEA cell migration in Replacement HGP and B. Desmoplastic HGP C. GSEA showing increased cell migration in Replacement HGP D. GSEA showing increased Adaptive immune response in dHGP and E. GSEA showing increased positive regulation of B cell activation in Desmoplastic.

|  |  |  |  |  |
| --- | --- | --- | --- | --- |
|  |  |  |  |  |
|  |  |  |  |  |
|  |  |  |  |  |
|  |  |  |  |  |
|  |  |  |  |  |
|  |  |  |  |  |
|  |  |  |  |  |
|  |  |  |  |  |
|  |  |  |  |  |
|  |  |  |  |  |
|  |  |  |  |  |
|  |  |  |  |  |
|  |  |  |  |  |
|  |  |  |  |  |
|  |  |  |  |  |
|  |  |  |  |  |
|  |  |  |  |  |
|  |  |  |  |  |
|  |  |  |  |  |
|  |  |  |  |  |
|  |  |  |  |  |
|  |  |  |  |  |

**S6 Table 2.** List of Diagnostic power by using ROC AUC analysis of all the differentiallyexpressed proteins.


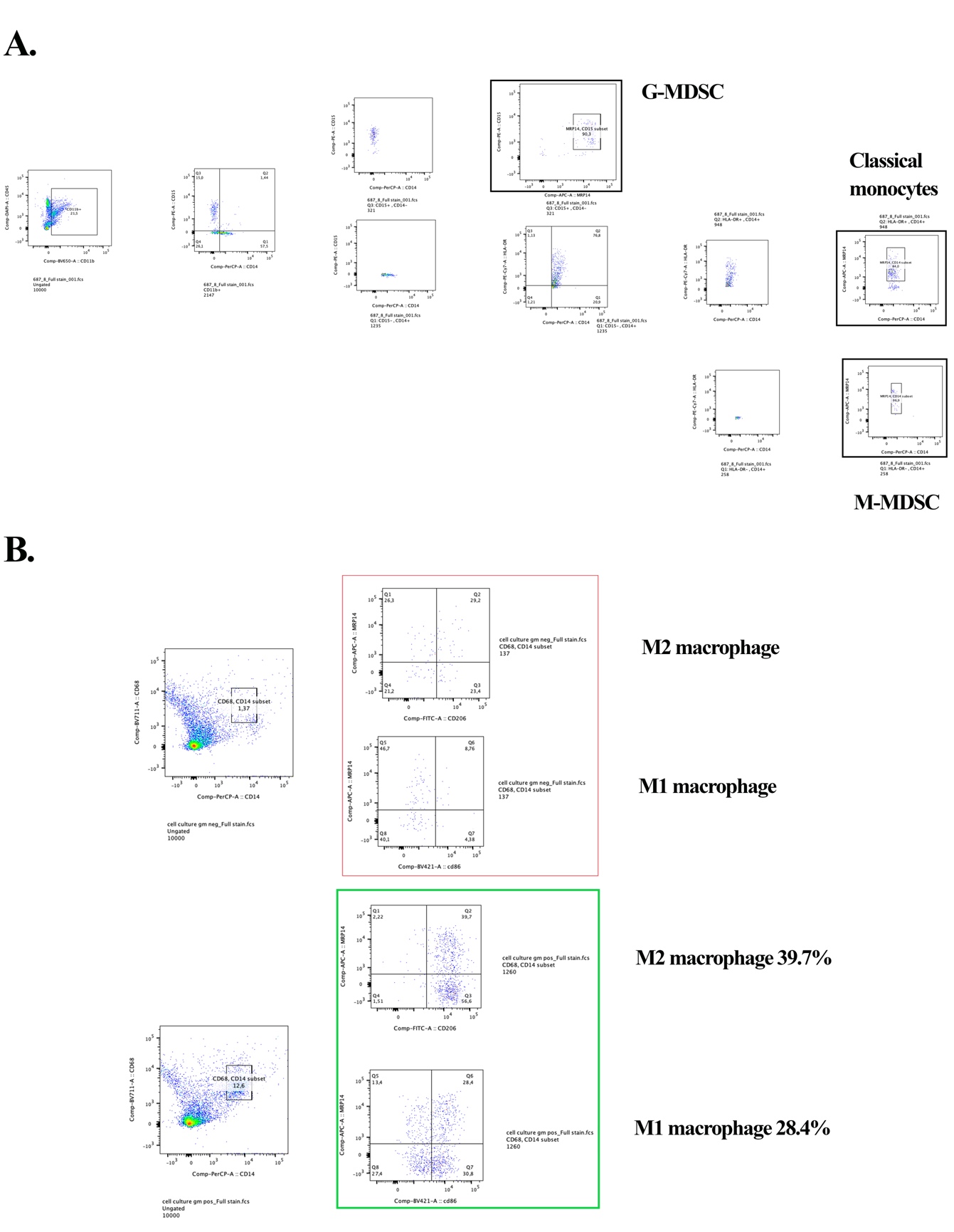


**S7 Figure: Characterization of Immune cells expressing S100A9**: S100A9 expressed by G-MDSC, M-MDSC and Classical monocytes expressing S100A9 B. Differentiated macrophage 28.4% of the macrophage population expressing S100A9 identified as M1 and 39.7% of the macrophage population expressing S100A9 identified as M2 without (Red box) and with GM-CSF treatment (Gr
